# Association Between Dinner-to-Bed Time and Gastroesophageal Reflux-Related Diseases in Children

**DOI:** 10.7759/cureus.77709

**Published:** 2025-01-20

**Authors:** Kensuke Uraguchi, Naomi Matsumoto, Toshiharu Mitsuhashi, Soshi Takao, Seiichiro Makihara, Mizuo Ando, Takashi Yorifuji

**Affiliations:** 1 Department of Epidemiology, Graduate School of Medicine, Dentistry and Pharmaceutical Sciences, Okayama University, Okayama, JPN; 2 Center for Innovative Clinical Medicine, Okayama University Hospital, Okayama, JPN; 3 Department of Otolaryngology - Head and Neck Surgery, Graduate School of Medicine, Dentistry and Pharmaceutical Sciences, Okayama University, Okayama, JPN; 4 Department of Otolaryngology- Head and Neck Surgery, Graduate School of Medicine, Dentistry and Pharmaceutical Sciences, Okayama University, Okayama, JPN

**Keywords:** acute otitis media, allergic rhinitis (ar), gastroesophageal reflux symptoms, pediatric asthma, public health

## Abstract

Introduction: Gastroesophageal reflux disease (GERD) is characterized by esophageal mucosal injury due to the reflux of gastroduodenal contents. Typical symptoms include heartburn and acid regurgitation. In addition, gastroesophageal reflux (GER) can influence conditions such as otitis media, rhinitis, and asthma. This study aimed to examine the association between dinner-to-bed time and GER-related diseases, such as otitis media, allergic rhinitis, and asthma.

Methods: This was a longitudinal cohort study using secondary data. Data were collected from a large-scale birth cohort study conducted in Japan including babies born in 2001 and 2010. Dinner-to-bed time was categorized as “longer dinner-to-bed time" (>120 minutes), “shorter dinner-to-bed time" (≤120 minutes or less), and “irregular dinner-to-bed time.” Modified Poisson regression with robust variance was used to estimate risk ratios (RRs).

Results: A total of 60,392 children were included in this study. Children with shorter dinner-to-bed time had a higher risk of asthma (adjusted RR (aRR) = 1.10; 95% confidence interval (CI), 1.03-1.18) than those with longer dinner-to-bed time. However, no significant association was observed between shorter dinner-to-bed time and otitis media or allergic rhinitis. Furthermore, supplementary analyses revealed that the risk of asthma was significantly higher in children born in 2001 (aRR = 1.13; 95% CI, 1.04-1.22).

Conclusion: This study showed that dinner-to-bed time within 120 minutes after dinner increases the risk of developing asthma. This underscores the importance of considering lifestyle modifications, as certain pediatric asthma cases may be influenced by behaviors that promote GER.

## Introduction

Gastroesophageal reflux disease (GERD) is a common disease characterized by esophageal mucosal injury secondary to the reflux of gastroduodenal contents into the esophagus. The most common symptoms of GERD are esophageal symptoms such as heartburn and regurgitation. However, in some cases, gastroesophageal reflux (GER) of stomach acid can cause extraesophageal symptoms such as asthma, chronic cough, and laryngitis. Furthermore, it can cause refractory and recurrent otitis media and rhinosinusitis when the reflux extends to the upper pharynx [1-3].

The prevalence of GERD has been increasing globally, and obesity and increased gastric acid secretion have been reported to be the primary contributors [4,5]. However, epidemiological research on GER/GERD in children has been largely limited to special cases or newborns, with few studies focusing on infants or school-aged children [6-9]. This may be because invasive diagnostic examinations for GERD, such as a pH/impedance study and endoscopy, are not recommended in pediatric populations without typical GERD symptoms of refractory GER-related diseases. Thus, the potential association between common pediatric conditions, such as otitis media, rhinitis, and asthma, and GERD remains largely unexplored.

In adults with GER/GERD, going to sleep shortly after dinner can increase the risk of GER-related diseases and exacerbate symptoms. Thus, it is generally avoided. However, the impact of this behavior on healthy children remains unclear [10]. The World Health Organization recommends that children aged three to four years get 10-13 hours of good quality sleep with regular sleep and wake-up times [11]. Thus, parents might inadvertently shorten the duration between dinner and bedtime for their children in an effort to ensure sufficient sleep.

This study aimed to investigate whether a short dinner-to-bed time interval increases the risk of GER-related diseases, such as otitis media, allergic rhinitis, and asthma. The results may provide parents with insights into reconsidering their lifestyle choices and help parents and pediatricians deepen their understanding of the disease.

## Materials and methods

Study design and participants

This was a cohort study using secondary data from a population-based birth cohort study in Japan, “The Longitudinal Survey of Babies in the 21st Century,” conducted by the Japanese Ministry of Health, Labor, and Welfare [12,13]. The Ethics Committee of Okayama University Graduate School of Medicine, Dentistry, and Pharmaceutical Sciences and Okayama University Hospital issued approval 1506-073. This study was conducted in accordance with the 2013 Declaration of Helsinki. Surveys were sent to households by post, requesting their participation in designing and implementing programs aimed at enhancing fertility, health, education, and other areas of concern. The survey was conducted in 2001 (2001 cohort) and 2010 (2010 cohort) to follow all babies born in Japan between January 10 and 17 or July 10 and 17, 2001, and between May 10 and 24, 2010. Baseline surveys (first surveys) were sent to all families when the babies reached six months of age. The surveys were sent to 53,575 families of babies born in 2001 and 43,767 families of babies born in 2010. Of the 53,575 and 43,767 questionnaires sent, 47,015 (88%) and 38,554 (88%) were completed and returned, and annual surveys were sent. A total of 65,471 children (38,961 born in 2001 and 26,510 born in 2010) whose families responded to the fifth survey and provided information on dinner and sleep times were enrolled in this study.

Dinner-to-bed time

Parents were asked about their usual dinner time on weekdays: "Did you eat at roughly the same time every day or at an irregular time (with a difference of more than an hour depending on the day)?" If the former, they were asked to specify their dining time in 10-minute increments. This information was collected when the infants were 4.5 years old, as reported in the fifth survey. In addition, parents were asked about their usual bedtime (see Appendix A). On the basis of the previous report, the dinner-to-bedtime interval, referred to as dinner-to-bed time, was calculated and categorized as “longer dinner-to-bed time" (>120 minutes) and “shorter dinner-to-bed time" (≤120 minutes). If the time for dinner or sleep was reported as irregular, it was categorized as “irregular dinner-to-bed time” [10].

GER-related diseases

The primary endpoint was to investigate GER-related diseases. Parents were asked if their children had received treatment for any diseases at hospitals or clinics within the past year and were asked to list all applicable diseases. From responses, otitis media, allergic rhinitis, and asthma were identified as GER-related diseases. Parents of children born in 2001 and 2010 reported on the same subject when the children were 5.5 years old on the sixth survey.

Statistical analysis

Dichotomous and categorical variables were presented as numbers and percentages, respectively. A comparison of the characteristics of children regarding dinner-to-bed time was made to assess the potential selection bias associated with loss to follow-up in the sixth survey.

Risk ratios (RRs) and 95% confidence interval (CI) were estimated using modified Poisson regression models with robust variance without offset terms for binary outcomes. Longer dinner-to-bed time (>120 minutes) was set as the reference category. Model 1 was a univariate analysis, and Model 2 was adjusted for child and parent factors related to lifestyle and GER.

**Figure 1:**
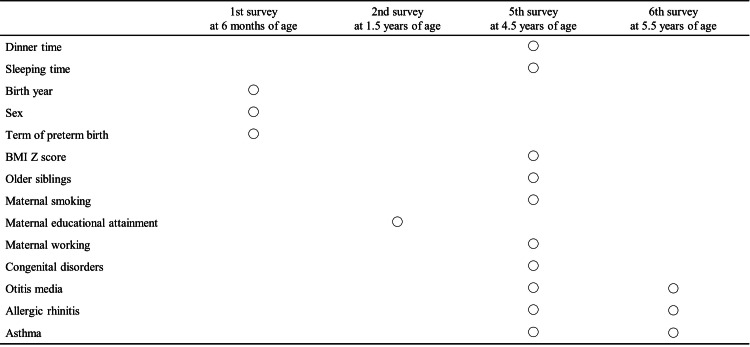
Measurement time point of survey data and question on children born in 2001 and 2010 BMI, body mass index Image credit: Kensuke Uraguchi

Based on a previous report, the following factors were selected as potential confounding factors (Figure 1). From the first survey at the age of 0.5 years, sex (male or female, dichotomous variable) and term or preterm birth (<37 weeks of gestation, dichotomous variable) were included. From the second survey at the age of 1.5 years, maternal educational attainment (junior high school or others, high school, junior college, or university or higher, categorical variable) was collected. From the fifth survey at the age of 4.5 years, other siblings (no other siblings, 1, or ≥2, categorical variable), including twin, maternal smoking (no smoking or present smoking, dichotomous variable), and maternal working (not working (no job, housemaker, or student), full-time job, or others (part-time job, self-employed, family business, side job, etc.), categorical variables) were included. The BMI Z score was calculated due to the association between GERD and obesity and classified into four groups (underweight, normal weight, overweight, and obese, categorical variable). Congenital disorders (yes or no, dichotomous variable) were considered a confounding factor because they have the potential to change lifestyle and are risk factors for GER.

Supplementary analysis was performed to assess effect heterogeneity, and two potential sources of variation were identified. The first was the birth year, reflecting the evolving landscape of disease diagnosis, prevention, and treatment over time. The second was the sleep time. The findings might be more influenced by the actual sleep time, whether earlier or not, than by the dinner-to-bedtime interval. Thus, a sleep time benchmark at 10 PM was established to delineate this. Stratified analyses were performed based on these factors.

Furthermore, restricted cubic splines with knots at the 10th, 50th, and 90th percentiles of the dinner-to-bed time distribution were used after excluding irregular dinner-to-bed time to evaluate nonlinear time-response variables. Using Model 2, the absolute risk and 95% CI for GER-related conditions were measured. A complete case analysis was performed. Statistical analyses were performed using Stata Statistical Software: release 18 (StataCorp, College Station, TX, USA). To ensure reproducibility of the study results, the Stata codes used in the study are presented in the Appendix (see Appendix B)

## Results

Participants

**Figure 2:**
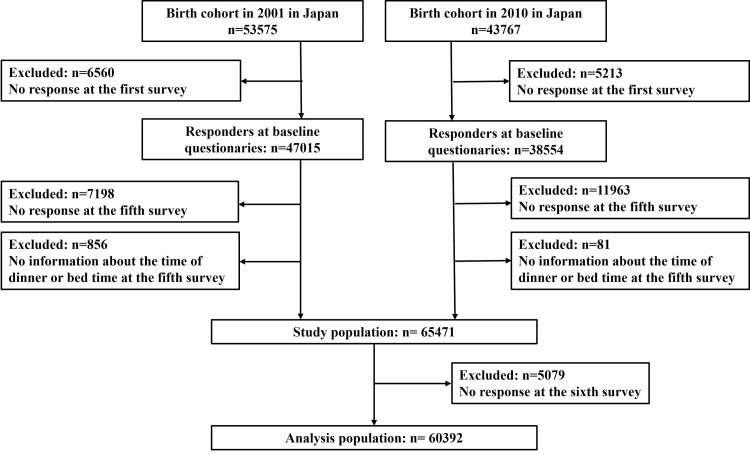
Study flowchart

A total of 65,471 families reported dinner-to-bed time. Among them, 5,079 were lost to follow-up due to nonresponse to the sixth survey. Finally, 60,392 children were included in this analysis (Figure 2).

Dinner-to-bed time

Table 1 shows the characteristics of the study participants. In the fifth survey at the age of 4.5 years, regarding dinner-to-bed time, 42,049 families reported “longer dinner-to-bed time" (>120 minutes), 17,890 reported “shorter dinner-to-bed time" (≤120 minutes), and 5,532 reported “irregular dinner-to-bed time.” Children with longer dinner-to-bed time had dinner earlier and slept later than those with shorter dinner-to-bed time.

**Table 1:** Characteristics of the study participants

Variables	Longer group (n = 42,049)	Shorter group (n = 17,890)	Irregular group (n = 5,532)
The time of dinner, n (%)			
Before 18	4,454 (11%)	464 (2.6%)	135 (2.4%)
18-19	24,359 (58%)	5,186 (29%)	1,169 (21%)
19-20	12,579 (30%)	10,753 (60%)	1,266 (23%)
After 20	657 (1.6%)	1,487 (8.3%)	163 (2.9%)
Irregular	0 (0.0%)	0 (0.0%)	2,799 (51%)
The time of sleeping, n (%)			
Before 21	5,172 (12%)	7,757 (43%)	157 (2.8%)
21-22	23,412 (56%)	9,255 (52%)	781 (14%)
22-23	11,778 (28%)	842 (4.7%)	641 (12%)
After 23	1,687 (4.0%)	36 (0.2%)	152 (2.7%)
Irregular	0 (0.0%)	0 (0.0%)	3,801 (69%)
Birth year, n (%)			
Birth in 2001	25,038 (60%)	11,223 (63%)	2,700 (49%)
Birth in 2010	17,011 (41%)	6,667 (37%)	2,832 (51%)
Gender, n (%)			
Male	21,888 (52%)	9,323 (52%)	2,836 (51%)
Female	20,161 (48%)	8,567 (48%)	2,696 (49%)
Term or preterm birth, n (%)			
Term (≥36week)	39,943 (95%)	17,001 (95%)	5,224 (94%)
Preterm(<36weeek)	2,106 (5.0%)	889 (5.0%)	308 (5.6%)
BMI Z score, n (%)			
Underweight	995 (2.4%)	408 (2.3%)	124 (2.2%)
Normal weight	31,584 (75%)	13,325 (75%)	3,992 (72%)
Overweight	4,559 (11%)	1,934 (11%)	571 (10%)
Obese	668 (1.6%)	282 (1.6%)	129 (2.3%)
Missing	4,243 (10%)	1941 (11%)	716 (13%)
Older siblings, n (%)			
No other siblings	8,230 (20%)	2,940 (16%)	1,424 (26%)
1	23,927 (57%)	10,458 (59%)	2,743 (50%)
≥2	9,892 (24%)	4,492 (25%)	1,365 (25%)
Maternal smoking, n (%)			
No smoking	36,021 (86%)	15,372 (86%)	4,435 (80%)
Present smoking	5,537 (13%)	2,273 (13%)	1,029 (19%)
Missing	491 (1.2%)	245 (1.4%)	68 (1.2%)
Maternal educational attainment, n (%)			
Junior high school or others	1,268 (3.0%)	478 (2.7%)	338 (6.1%)
High school	14,034 (33%)	5,555 (31%)	2,251 (41%)
Junior college	17,179 (41%)	7,675 (43%)	1,976 (36%)
University or higher	8,415 (20%)	3,689 (21%)	789 (14%)
Missing	1,153 (2.7%)	493 (2.8%)	178 (3.2%)
Maternal working, n (%)			
Not working	18,770 (45%)	9,370 (52%)	2,498 (45%)
Full-time job	8,842 (21%)	3,049 (17%)	996 (18%)
Others	13,841 (33%)	5,151 (29%)	1,960 (35%)
Missing	596 (1.4%)	320 (1.8%)	78 (1.4%)
Congenital disorders, n (%)			
No	41,605 (99%)	17,727 (99%)	5,458 (99%)
Yes	444 (1.1%)	163 (0.9%)	74 (1.3%)

Children lost to follow-up tended to eat dinner and sleep later, the number of those born in 2010 was larger, and the proportion of mothers who were smokers was higher. However, no significant differences in dinner-to-bed time were observed between children lost to follow-up and those included in the final analysis (Table 2).

**Table 2:** Characteristics of children lost to follow-up and those included in the analysis BMI: body mass index

Variables	Analyzed data (n = 60,392)	Loss to follow-up (n = 5,079)
Dinne-to-bed time, n (%)		
Longer group	38,946 (65%)	3,103 (61%)
Shorter group	16,483 (27%)	1,407 (28%)
Irregular group	4,963 (8.2%)	569 (11%)
The time of dinner, n (%)		
Before 18	4,742 (7.9%)	311 (6.1%)
18-19	28,523 (47%)	2,191 (43%)
19-20	22,570 (37%)	2,028 (40%)
After 20	2,064 (3.4%)	243 (4.8%)
Irregular	2,493 (4.1%)	306 (6.0%)
The time of sleeping, n (%)		
Before 21	12,211 (20%)	875 (17%)
21-22	31,006 (51%)	2,442 (48%)
22-23	12,108 (20%)	1,153 (23%)
After 23	1,658 (2.7%)	217 (4.3%)
Irregular	3,409 (5.6%)	392 (7.7%)
Birth year, n (%)		
Birth in 2001	36,521 (61%)	2,440 (48%)
Birth in 2010	23,871 (40%)	2,639 (52%)
Gender, n (%)		
Male	31,369 (52%)	2,678 (53%)
Female	29,023 (48%)	2,401 (47%)
Term or preterm birth, n (%)		
Term (≥36 week)	57,357 (95%)	4,811 (95%)
Preterm(<36 weeek)	3,035 (5.0%)	268 (5.3%)
BMI Z score, n (%)		
Underweight	1,392 (2.3%)	135 (2.7%)
Normal weight	45,423 (75%)	3,478 (69%)
Overweight	6,532 (11%)	532 (11%)
Obese	977 (1.6%)	102 (2.0%)
Missing	6,068 (10%)	832 (16%)
Older siblings, n (%)		
No other siblings	11,644 (19%)	950 (19%)
1	34,425 (57%)	2,703 (53%)
≥2	14,323 (24%)	1,426 (28%)
Maternal smoking, n (%)		
No smoking	51,889 (86%)	3,939 (78%)
Present smoking	7,788 (13%)	1,051 (21%)
Missing	715 (1.2%)	89 (1.8%)
Maternal educational attainment, n (%)		
Junior high school or others	1,791 (3.0%)	293 (5.8%)
High school	20,027 (33%)	1,813 (36%)
Junior college	25,053 (42%)	1,777 (35%)
University or higher	12,119 (20%)	774 (15%)
Missing	1,402 (2.3%)	422 (8.3%)
Maternal working, n (%)		
Not working	28,624 (47%)	2,014 (40%)
Full-time job	11,820 (20%)	1,067 (21%)
Others	19,066 (32%)	1,886 (37%)
Missing	882 (1.5%)	112 (2.2%)
Congenital disorders, n (%)		
No	59,758 (99%)	5,032 (99%)
Yes	634 (1.0%)	47 (0.9%)

Association between dinner-to-bed time and GER-related diseases

Table 3 shows the Poisson regression with robust variance between dinner-to-bed time and GER-related diseases. The multivariate analysis revealed that shorter dinner-to-bed time was associated with a higher risk of asthma (adjusted RR (aRR) = 1.10; 95% CI, 1.03-1.18). However, no significant association was observed between shorter dinner-to-bed time and otitis media and allergic rhinitis.

**Table 3:** Univariate and multivariate analyses of the association between dinner-to-bed time and GER-related diseases RR: risk ratio, CI: confidence interval Model 1: univariate analysis; Model 2: multivariate analysis adjusted with children and parent factor

Outcomes	Cases (%)	Model 1: RR (95% CI)	Model 2: Adjusted RR (95% CI)
Otitis media			
More than 120 minutes	6,016 (16%)	1 (Reference)	1 (Reference)
120 minutes or less	2,576 (16%)	1.01 (0.97–1.06)	1.00 (0.96–1.05)
Irregular	783 (16%)	1.02 (0.95–1.09)	1.02 (0.94–1.09)
Allergic rhinitis			
More than 120 min	4,794 (12%)	1 (Reference)	1 (Reference)
120 minutes or less	2,061 (13%)	1.02 (0.97–1.07)	1.02 (0.97–1.07)
Irregular	576 (12%)	0.94 (0.87–1.02)	0.96 (0.88–1.04)
Asthma			
More than 120 minutes	3,042 (7.8%)	1 (Reference)	1 (Reference)
120 minutes or less	1,393 (8.5%)	1.08 (1.02–1.15)	1.10 (1.03–1.18)
Irregular	404 (8.1%)	1.04 (0.94–1.15)	0.99 (0.89–1.10)

In the supplementary analysis, children were stratified according to birth year and sleep time before or after 10 PM (Table 4). The analysis revealed that an increased risk of asthma was modified by the birth year 2001 (aRR = 1.13; 95% CI, 1.04-1.22) and sleeping before 10 PM (aRR = 1.08; 95% CI, 1.01-1.16).

**Table 4:** Stratified by birth year and sleeping time RR: risk ratio, CI: confidence interval, N/A: not applicable Adjusted with children and parent factor as Model 2

Outcomes	Otitis media		Allergic rhinitis		Asthma	
	Cases (%)	Adjusted RR (95% CI)	Cases (%)	Adjusted RR (95% CI)	Cases (%)	Adjusted RR (95% CI)
Born in 2010						
More than 120 minutes	3,762 (16%)	1 (Reference)	3128 (13%)	1 (Reference)	1940 (8.2%)	1 (Reference)
120 minutes or less	1,679 (16%)	0.99 (0.93–1.04)	1404 (13%)	1.01 (0.95–1.08)	965 (9.2%)	1.13 (1.04–1.22)
Irregular	391 (16%)	1.00 (0.90–1.11)	309 (13%)	0.97 (0.87–1.09)	224 (9.1%)	1.06 (0.92–1.22)
Born in 2010						
More than 120 minutes	2,254 (15%)	1 (Reference)	1666 (11%)	1 (Reference)	1102 (7.2%)	1 (Reference)
120 minutes or less	897 (15%)	1.02 (0.95–1.11)	657 (11%)	1.01 (0.92–1.11)	428 (7.2%)	1.03 (0.92–1.16)
Irregular	392 (16%)	1.05 (0.95–1.17)	267 (11%)	0.99 (0.87–1.13)	180 (7.2%)	0.94 (0.79–1.12)
Sleep before PM10						
More than 120 minutes	4,216 (16%)	1 (Reference)	3403 (13%)	1 (Reference)	2089 (7.8%)	1 (Reference)
120 minutes or less	2,463 (16%)	0.99 (0.94–1.04)	1,986 (13%)	0.99 (0.94–1.05)	1329 (8.5%)	1.08 (1.01–1.16)
Irregular	N/A	N/A	N/A	N/A	N/A	N/A
Sleep after PM10						
More than 120 minutes	1,800 (15%)	1 (Reference)	1391 (11%)	1 (Reference)	953 (7.8%)	1 (Reference)
120 minutes or less	113 (14%)	0.97 (0.80–1.17)	75 (9.5%)	0.88 (0.70–1.12)	64 (8.1%)	1.19 (0.92–1.53)
Irregular	N/A	N/A	N/A	N/A	N/A	N/A

Restricted cubic spline

**Figure 3:**
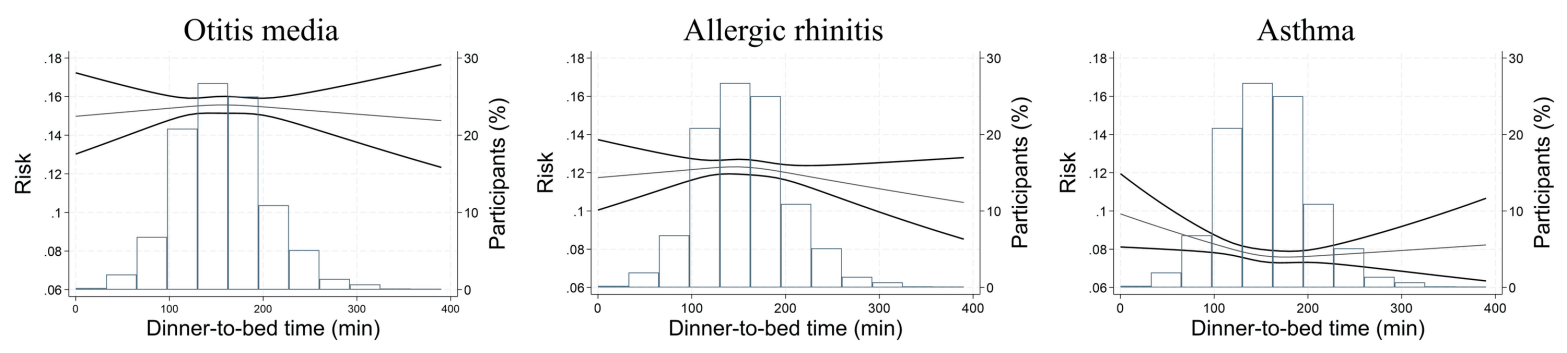
Risk of GER-related diseases by dinner-to-bed time in restricted cubic spline. The graph plots absolute risk on the left axis and dinner-to-bed time on the x-axis, with participant percentages on the right axis. In addition, the graph indicates the percentage of participants for each dinner-to-bed time, which is presented on the right axis.

The restricted cubic spline regression analyses revealed that longer dinner-to-bed time was associated with a decreased risk of asthma. However, dinner-to-bed time did not affect the risk of otitis media and allergic rhinitis (Figure 3).

## Discussion

This study showed that shorter dinner-to-bed time increased the risk of asthma. However, no association was observed with the risk of otitis media or allergic rhinitis. These results are anatomically plausible with respect to the progression of acid reflux. These findings highlight that lifestyle factors related to GER could contribute to the onset of common pediatric diseases, suggesting the need for future considerations of lifestyle modifications.

GER is a normal physiological process found in healthy individuals, from children to adults, and it may not always lead to symptoms or complications. With the presence of symptoms or complications due to reflux of gastroduodenal contents, GERD occurs [14]. Few large population-based studies have been conducted on pediatric GER/GERD. Moreover, the prevalence is age-dependent. The prevalence of GER/GERD in children aged less than one year was high (59% and 19%), but most cases resolved on their own [9,15]. Previous epidemiological studies on children aged three to 17 years reported a GERD prevalence rate of 1.8-8.2% [16]. The prevalence then rises with age, reaching adult levels by adolescence [7]. Recently, the prevalence of GERD has been reported to increase in children, which may be influenced by factors such as an increase in stomach acid secretion and a high-protein diet [4].

Lifestyle plays a significant role in GER/GERD. Thus, lifestyle modifications are recommended, including weight loss, elevation of the head of the bed, avoidance of lying down immediately after meals, and avoidance of dietary triggers and bedtime snacks. Many of these recommendations were based on studies on adults, and evidence for some interventions is limited [14]. Focusing on dinner-to-bed time, a previous randomized controlled trial conducted on adults with GERD reported that dinner-to-bed time of two hours was associated with an increase in supine reflux and an increase in the number of individuals reporting heartburn as their primary complaint compared with dinner-to-bed time of six hours [10]. In addition, a study conducted in Japan showed that a dinner-to-bed time of less than three hours was associated with an increased risk of GERD compared with a dinner-to-bed time of four hours or more [17]. However, according to the American College of Gastroenterology guidelines, early bedtime after dinner for adults was still given a “low evidence and conditional recommendation” [18]. However, this study revealed an association between early bedtime and the onset of GER-related asthma in children. This finding suggests that future lifestyle modifications help prevent certain cases of asthma.

GERD is recognized not only by its typical esophageal symptoms, such as heartburn and acid regurgitation, but also by its potential to influence various diseases through extraesophageal presentations caused by GER [19]. Particularly, many reports on asthma have been made, and the prevalence of GERD in pediatric patients with asthma has been reported to be 22%, suggesting that GER might be a trigger for exacerbation [20]. The mechanisms by which GER/GERD causes asthma include the reflex theory, where acid stimulates the vagal receptors in the lower esophagus, reflexively stimulating the lower airways, and the microaspiration theory, where the refluxed content reaches the pharynx and larynx, leading to aspiration and irritation [21,22]. Furthermore, studies have suggested that reflux from the nasopharynx to the eustachian tube and nasal cavity can cause otitis media and rhinosinusitis [23-25]. The presence of pepsin or pepsinogen in the middle ear may be a marker for physiologic reflux events [26]. GERD is also hypothesized to contribute to otitis media with effusion, but causality has not been proven, and findings are inconsistent [27]. When refractory otitis media, rhinitis, and asthma are associated with extraesophageal presentations due to GERD, proton pump inhibitors are sometimes considered effective treatment for GERD-related symptoms [28,29]. However, this perspective remains a matter of debate. Some randomized controlled trials have reported no discernible benefits [30,31]. In addition to the possibility of being ineffective for these conditions, the use of proton pump inhibitors could potentially heighten the risk of future allergic diseases. Thus, the decision to administer them should be made with caution [32,33].

The major strength of this study is that it is a large population-based study that focuses on children born in 2001 and 2010. Previous studies have relied only on case series or case-control studies to investigate the relationship between children’s lifestyles and GER-related diseases. In addition, we adjusted for key GER risk factors, including congenital disorders, obesity, and maternal smoking. Although our initial hypothesis suggested a consistent effect of bedtime after dinner on GER/GERD across various eras, supplementary analysis indicated an increased risk of developing asthma only in the 2001-born cohort. This may be due to the enhanced prevention and control measures for pediatric asthma in Japan over the past decade, resulting in a decline in the prevalence rate [34]. Consequently, the potential influence of GER on the onset of asthma appears to be minimal. Understanding the implications of lifestyle factors (e.g., dinner-to-bed time), which are universally applicable to all children is crucial for informing public health strategies. Even slight increases in risks, when considered across a population level, underscore the importance of addressing these modifiable behaviors.

This study has some limitations. First, the survey did not include questions about typical GERD symptoms, such as heartburn and acid reflux, because this research utilized secondary data not originally collected for research purposes. Therefore, whether GERD is truly involved is unclear. Furthermore, this study investigated lifestyles related to GER-related diseases, but the actual presence or absence of GER/GERD was unclear. Second, this study did not clarify the presence or absence of night snacking after dinner. Third, the prevalence of GER/GERD varies by age, and the long-term effects are unclear. Therefore, the study findings may not be generalized to all children. Fourth, the outcomes were reported by parents based on the doctor’s diagnosis. Therefore, there is a possibility of measurement bias. In addition, data on diagnosis, severity, or treatment were unknown. Hence, cases with mild symptoms, which do not necessitate medical consultation, might not have been diagnosed, thereby potentially underestimating the diagnosis. Furthermore, the definitions of diagnoses have not been validated. For example, children with allergic rhinitis and asthma could include nonallergic cases. Moreover, in cases of otitis media, the distinctions between acute otitis media, serous otitis media, and cholesteatoma were not made. However, most cases of otitis media in children are acute otitis media, and serous otitis media often arises from acute otitis media. Therefore, otitis media can be an approximation of acute otitis media. Fifth, unmeasured confounders are possible. GER/GERD and GER-related diseases such as otitis media, allergic rhinitis, and asthma are multifactorial and not solely influenced by lifestyle. For example, the prevalence of cow’s milk allergy and functional gastrointestinal disorders, which are associated with GERD and allergic diseases, needs to be explored [35,36]. Sixth, due to sample size imbalances and differences in prevalence observed in the stratification of supplementary analyses, accurate estimation of effect heterogeneity may not have been achieved, warranting caution in interpretation.

## Conclusions

Early bedtime after dinner may increase the risk of developing asthma, a GER-related disease, in children. Certain cases of pediatric asthma may be related to GER-promoting lifestyles. Thus, lifestyle modifications should be considered. Further prospective studies employing specific questions and examinations are warranted to elucidate the environmental factors contributing to GER/GERD.

## Appendices

Appendix A: survey questions focusing on children born in 2001 and 2010

1)      Dinner time (fifth survey)

When does your child usually have dinner?

1.      Around a fixed time (around __:__0)

2.      Irregular time (varies by more than an hour depending on the day)

3.      Does not eat dinner

2)      Sleeping time (fifth survey)

When does your child go to sleep at night?

1.      Around a fixed time (around __:__0)

2.      Irregular time (varies by more than an hour depending on the day)

3)      Other siblings (fifth survey)

Who does your child live with? Number of siblings: __

4)      Maternal smoking (fifth survey)

Does the mother usually smoke?

5)      Maternal educational attainment (second survey)

Please tell us about the last school the mother graduated from or is attending. Please mark the applicable number.

1.      Junior high school

2.      Upper secondary specialized training school

3.      High school

4.      Professional training college

5.      Junior college

6.      University

7.      Graduate school

8.      Others

6)      Maternal working (fifth survey)

Please mark the number corresponding to the mother's employment status.

1.      Housemaker

2.      No job

3.      Student

4.      Full-time job

5.      Part-time job

6.      Self-employed/family business

7.      Side job

8.      Other

7)      Yearly medical visits for an illness or injury (fifth and sixth surveys)

Have you been examined by a physician at a hospital or clinic for an illness or injury in the past year? Please mark all applicable numbers (multiple choice).

01.   Nothing

02.   Varicella

03.   Measles

04.   Rubella

05.   Mumps

06.   Kawasaki disease

07.   Allergic rhinitis/allergic conjunctivitis

08.   Asthma

09.   Atopic dermatitis

10.   Food allergy

11.   Conjunctivitis

12.   Otitis media

13.   Otitis externa

14.   Cold, bronchitis, bronchiolitis, and pneumonia

15.   Influenza

16.   Gastrointestinal diseases

17.   Impetigo

18.   Eczema

19.   Congenital diseases

20.   Seizures

21.   Dental caries

22.   Consultation for development and behavior

23.   Pharyngoconjunctival fever

24.   Streptococcal infection

25.   Other diseases

26.   Injuries (including fractures and burns)

Appendix B: statistical code for Stata (version 18)

*Independent variable:

*sleeping120 (Dinner-to-bed time at 5th survey; 0: More than 120 min, 1: 120 min or less, 2: Irregular)

* Dependent variables:

*1) om6 (Otitis media at 6th survey; 0: No, 1: Yes)

*2) ar6 (Allergic rhinitis at 6th survey; 0: No, 1: Yes)

*3) asthma6 (Asthma at 6th survey; 0: No, 1: Yes)

* Adjusted variables:

*1) sex (Sex; 0: Female, 1: Male)

*2) week (Term or preterm birth; 0: Term (≥36 weeks), 1: Preterm (<36 weeks))

 *3) bmiz5 (BMI Z score at 5th survey; 0: Underweight, 1: Normal weight, 2: Overweight, 3: Obese)

 *4) sibling5 (Older siblings at 5th survey; 0: No siblings, 1: 1 sibling, 2: ≥2 siblings)

 *5) msmoking5 (Maternal smoking at 5th survey; 0: No smoking, 1: Present smoking)

 *6) meducation2 (Maternal educational attainment at 2nd survey; 0: Junior high school or others, 1: High school, 2: Junior college, 3: University or higher)

 *7) mworking5 (Maternal working at 5th survey; 0: Not working, 1: Full-time job, 2: Others)

 *8) congenital5 (Congenital diseases at 5th survey; 0: No, 1: Yes)

*Table 2: Association between Dinner-to-Bed Time and GER-related Diseases

*Model 1: Crude analysis

glm om6 i.sleeping120 ,fam(poisson) link(log) nolog vce(robust)eform

glm ar6 i.sleeping120,fam(poisson) link(log) nolog vce(robust)eform

glm asthma6 i.sleeping120 ,fam(poisson) link(log) nolog vce(robust)eform

*Model 2: Adjusted analysis

glm om6 i.sleeping120 sex week bmiz5 sibling5 msmoking5 meducation2 mworking5 congenital5, fam(poisson) link(log) nolog vce(robust)eform

glm ar6 i.sleeping120 sex week bmiz5 sibling5 msmoking5 meducation2 mworking5 congenital5, fam(poisson) link(log) nolog vce(robust)eform

glm asthma6 i.sleeping120 sex week bmiz5 sibling5 msmoking5 meducation2 mworking5 congenital5, fam(poisson) link(log) nolog vce(robust)eform

*TABLE 4: Stratified by Birth Year and Sleeping Time

*Birth year (0: Born in 2001, 1: Born in 2010)

bysort year:glm om6 i.sleeping120 sex week bmiz5 sibling5 msmoking5 meducation2 mworking5 congenital5, fam(poisson) link(log) nolog vce(robust)eform

bysort year:glm ar6 i.sleeping120 sex week bmiz5 sibling5 msmoking5 meducation2 mworking5 congenital5, fam(poisson) link(log) nolog vce(robust)eform

bysort year:glm asthma6 i.sleeping120 sex week bmiz5 sibling5 msmoking5 meducation2 mworking5 congenital5, fam(poisson) link(log) nolog vce(robust)eform

*Sleeping time (0: Sleep before PM10, 1: Sleep after PM10)

bysort pm22:glm om6 i.sleeping120 sex week bmiz5 sibling5 msmoking5 meducation2 mworking5 congenital5, fam(poisson) link(log) nolog vce(robust)eform

bysort pm22:glm ar6 i.sleeping120 sex week bmiz5 sibling5 msmoking5 meducation2 mworking5 congenital5, fam(poisson) link(log) nolog vce(robust)eform

bysort pm22:glm asthma6 i.sleeping120 sex week bmiz5 sibling5 msmoking5 meducation2 mworking5 congenital5, fam(poisson) link(log) nolog vce(robust)eform

FIGURE 3: Risk of GER-related Diseases by Dinner-to-Bed Time in Restricted Cubic Spline.

*Otitis media

mkspline sleeping_time_sp = sleeping_time, nknots(3) cubic

glm om6 sleeping_time_sp* sex week bmiz5 sibling5 msmoking5 meducation2 mworking5 congenital5, fam(poisson) link(log) nolog vce(robust) eform

foreach v of varlist sex week sibling5 msmoking5 mworking5 bmiz5 congenital5 meducation{

                  sum `v'

                  gen     `v'_old = `v'

                  replace `v'=r(mean)

}

levelsof sleeping_time

predictnl ir= xb(), ci(lb ub)

replace ir=exp(ir)

replace lb=exp(lb)

replace ub=exp(ub)

twoway line ir sleeping_time

sort sleeping_time

keep if sleeping_time<400

twoway ///

                  (line ub sleeping_time, bcolor(gs14) lwidth(medthick) lcolor(black)) ///

                  (line lb sleeping_time, bcolor(gs14) lwidth(medthick) lcolor(black)) ///

                  (line ir sleeping_time, lpattern(solid) lcolor(gs5)) ///

                  (hist sleeping_time, percent bin(12) fcolor(none) lcolor(edkblue) lw(medthin) yaxis(2)), ///

                                   ytitle("Risk", axis(1)) xtitle("Dinner-to-bed time (min)") ///

                                   legend(off) ///

                                   ytitle("(%)", axis(2)) ///

                                   xscale(range (0 300) ) yscale(range(0.06 0.18) axis(1)) yscale(range(0 30) axis(2))

*Allergic rhinitis

mkspline sleeping_time_sp = sleeping_time, nknots(3) cubic

glm ar6 sleeping_time_sp* sex week bmiz5 sibling5 msmoking5 meducation2 mworking5 congenital5, fam(poisson) link(log) nolog vce(robust) eform

foreach v of varlist sex week sibling5 msmoking5 mworking5 bmiz5 congenital5 meducation{

                  sum `v'

                  gen     `v'_old = `v'

                  replace `v'=r(mean)

}

levelsof sleeping_time

predictnl ir= xb(), ci(lb ub)

replace ir=exp(ir)

replace lb=exp(lb)

replace ub=exp(ub)

twoway line ir sleeping_time

sort sleeping_time

keep if sleeping_time<400

twoway ///

                  (line ub sleeping_time, bcolor(gs14) lwidth(medthick) lcolor(black)) ///

                  (line lb sleeping_time, bcolor(gs14) lwidth(medthick) lcolor(black)) ///

                  (line ir sleeping_time, lpattern(solid) lcolor(gs5)) ///

                  (hist sleeping_time, percent bin(12) fcolor(none) lcolor(edkblue) lw(medthin) yaxis(2)), ///

                                   ytitle("Risk", axis(1)) xtitle("Dinner-to-bed time (min)") ///

                                   legend(off) ///

                                   ytitle("(%)", axis(2)) ///

                                   xscale(range (0 300) ) yscale(range(0.06 (0.02) 0.18) axis(1))yscale(range(0 30) axis(2))

*Asthma

mkspline sleeping_time_sp = sleeping_time, nknots(3) cubic

glm ar6 sleeping_time_sp* sex week bmiz5 sibling5 msmoking5 meducation2 mworking5 congenital5, fam(poisson) link(log) nolog vce(robust) eform

foreach v of varlist sex week sibling5 msmoking5 mworking5 bmiz5 congenital5 meducation{

                  sum `v'

                  gen     `v'_old = `v'

                  replace `v'=r(mean)

}

levelsof sleeping_time

predictnl ir= xb(), ci(lb ub)

replace ir=exp(ir)

replace lb=exp(lb)

replace ub=exp(ub)

twoway line ir sleeping_time

sort sleeping_time

keep if sleeping_time<400

twoway ///

                  (line ub sleeping_time, bcolor(gs14) lwidth(medthick) lcolor(black)) ///

                  (line lb sleeping_time, bcolor(gs14) lwidth(medthick) lcolor(black)) ///

                  (line ir sleeping_time, lpattern(solid) lcolor(gs5)) ///

                  (hist sleeping_time, percent bin(12) fcolor(none) lcolor(edkblue) lw(medthin) yaxis(2)), ///

                                   ytitle("Risk", axis(1)) xtitle("Dinner-to-bed time (min)") ///

                                   legend(off) ///

                                   ytitle("(%)", axis(2)) ///

                                   xscale(range (0 300) ) yscale(range(0.06 (0.02) 0.18) axis(1))yscale(range(0 30) axis(2))
